# Fabrication of a Superhydrophobic Surface via Wet Etching of a Polydimethylsiloxane Micropillar Array

**DOI:** 10.3390/polym18010132

**Published:** 2025-12-31

**Authors:** Wu-Hsuan Pei, Chuan-Chieh Hung, Yi-Je Juang

**Affiliations:** 1 Department of Chemical Engineering, National Cheng Kung University, Tainan 70101, Taiwan; 2 Core Facility Center, National Cheng Kung University, Tainan 70101, Taiwan

**Keywords:** superhydrophobic surface, micropillar array, polydimethylsiloxane (PDMS), Cassie–Baxter state, wet etching

## Abstract

Superhydrophobic surfaces have gained considerable attention due to their ability to repel water and reduce surface adhesion, and they are now widely applied for self-cleaning, anti-fouling, anti-icing, and corrosion resistance purposes. In this study, either a computer numerical control (CNC) machine or photolithographic techniques were employed to fabricate molds with microwells, followed by soft lithography to obtain a polydimethylsiloxane (PDMS) micropillar array. An etching process was then carried out. It was found that, as etching time increased, the diameters of micropillars decreased, leading to a decrease in the solid fraction of the composite surface and increases in contact angles. When the ratios of spacing to diameter (W/D) and of height to diameter (H/D) both exceeded 1.5, the contact angle was found to exceed 150° and the original PDMS micropillar surface with a contact angle of around 135° became superhydrophobic. A drastic decrease in sliding angle was also observed at this threshold. Changes in contact angles with different W/D values were in good agreement with values calculated using the Cassie–Baxter equation, and the droplet state was verified by a pressure balance model. Meanwhile, the PDMS etching rate when using acetone as the solvent was approximately 6–8 times faster than that when using 1-Methyl-2-pyrrolidone (NMP), a result which is comparable to data in the literature.

## 1. Introduction

Superhydrophobic surfaces, due to their unique water-repulsion characteristics, have been utilized in many areas, such as self-cleaning [1], oil–water separation [2], anti-corrosion [3], anti-icing [4], and microfluidic systems [5]. Fabrication of superhydrophobic surfaces fundamentally relies on two key factors: reduction in surface free energy, and enhancement of surface roughness [6,7,8,9,10]. The former is dictated by the intrinsic physicochemical properties of the substrate material, whereas the latter is typically achieved through engineering of micro- or nanoscale surface topographies so that they resemble hierarchical structures found in natural systems, such as the nanofibers on lotus leaves [11] or the setae on gecko feet [12]. The introduction of surface roughness enhances hydrophobicity by increasing the effective surface area and generating air pockets within the surface features. These air pockets lead to formation of a composite solid–air–liquid interface, in contrast to a homogeneous solid–liquid interface [7,13,14]. In addition to geometric considerations and surface properties, the size of the water droplet also plays a role in wetting behavior [15], and the balance between the Laplace pressure of the droplet and the capillary pressure of the surface has been proposed to elucidate the mechanism behind the Cassie–Wenzel transition [16,17].

Myriads of methods to fabricate superhydrophobic surfaces have been proposed and demonstrated. In general, these can be categorized into two categories. One category of methods involves producing surfaces with irregular topography, the other involves producing surfaces with regular topography. Regarding the former category, a vast variety of techniques can be found in the literature; these include chemical etching, plasma etching, layer-by-layer deposition, and sol–gel processing, as well as spray, spin, and dip coating [18,19]. When methods from the latter category are used, surfaces consisting of regularly spaced micropillars or microwells are constructed. Although generating irregular surface topography is usually a low-cost, rapid, and scalable method which produces mechanically durable results, micropillar arrays offer precise, reproducible, and tunable surface engineering with topographic design principles to regulate interfacial phenomena across multiple length scales [20]. Kwon et al. fabricated microscale pillar structures on silicon substrates using deep reactive-ion etching (DRIE) followed by xenon difluoride (XeF_2_) etching to introduce nanoscale surface roughness. This hierarchical structure increased the water contact angle to 173°, so that a Cassie–Baxter state was exhibited in which the water droplet was suspended on the top of the pillars, without contacting the substrate [21]. Yin et al. employed laser-induced deep etching (LIDE) and chemically wet etching to fabricate tapered microholes on fused silica substrates, followed by PDMS replication and silanization to obtain conical micropillar arrays from which a superhydrophobic surface was obtained [22]. Kim et al. fabricated hierarchical micro/nanoscale mushroom-shaped structures by first performing reactive-ion etching (RIE) on a silicon substrate, followed by XeF_2_ etching and subsequent replication. This process transformed originally hydrophilic polyurethane (PU) into a superhydrophobic surface with self-cleaning properties [23]. Kim and Park applied photolithography to generate hydrogel micropillars whose tops were capped with PDMS [24]. The surface showed antifogging and self-cleaning performance, with an advancing contact angle of 166° and a receding contact angle of 131°. Zgaren et al. utilized liquid-crystal display (LCD) 3D printing technology to create micropillars with various geometries, including square, cylindrical, hexagonal, and truncated conical forms [25]. With surface modification, the pillar array exhibited an anti-icing property. Zhang et al. used a femtosecond laser to create microholes on a polytetrafluoroethylene (PTFE) film, then spin-coated a mixture of PDMS, curing agent, and carbon powder on the mold to create micropillars with a contact angle of around 150° [26]. In [27], photolithography and inductively coupled plasma etching were used to generate silicon micropillars, followed by chemical modification to obtain a superhydrophobic surface for subsequent investigation of the freezing behavior and ice-adhesion properties of sessile drops. In [28], a superhydrophobic surface was constructed by generating micro/nanoscale hierarchical micropillar arrays via ultrasonic plasticizing and pressing, with 304 stainless-steel thin sheets with micron-scale through-holes used as primary templates, and anodic aluminum oxides (AAOs) with ordered nanoscale pores used as secondary templates [28]. In [29], micropillars were fabricated on biaxially oriented polystyrene films using a picosecond laser in conjunction with chemical modification, to yield superhydrophobic surfaces with a contact angle larger than 160°.

Conventionally, the characteristic dimensions of nanopillars or micropillars on a superhydrophobic surface often range from hundreds of nanometers to tens of microns, so that photolithography or even more costly processes are required. In order to circumvent the photolithographic process, Deng and Juang exploited CNC micromachining to fabricate an array of microwells with diameters up to hundreds of microns. The microwells were then casted with polydimethylsiloxane (PDMS) to form micropillars; these then underwent a wet etching process [30]. In this study, the hydrophobicity of a surface with etched micropillars was investigated. First, PDMS-based micropillar arrays with feature sizes ranging from several tens to hundreds of micrometers were fabricated, either through photolithography or CNC micromachining; these then underwent a wet etching process. In addition to assessing the hydrophobicity of the surface, a particular focus was on elucidating relationship between microstructural parameters and superhydrophobicity.

## 2. Materials and Methods

### 2.1. Materials

Silicon wafer (Si, N-Type) was purchased from Ultimate Materials Technology, Co., Ping Tung, Taiwan. Negative photoresist (SU-8 2050) and developer (Propylene glycol monomethyl ether acetate, PGMEA) were purchased from Micro-Chem, Adel, GA, USA. Polydimethylsiloxane (PDMS, Sylgard 184) was purchased from Dow Corning, Midland, MI, USA. PDMS etchant (75% (*w*/*v*) tetra-n-butylammonium fluoride (TBAF) in water) and 1-Methyl-2-pyrrolidone (NMP) were purchased from Alfa Aesar, Ward Hill, MA, USA. Acetone was purchased from J. T. Baker, Phillipsburg, NJ, USA.

### 2.2. Fabrication of Micropillar Array via Photolithography

A photoresist microwell array was first obtained through standard photolithography. The diameters of the microwells were 50 or 100 μm, and their depth was 100 μm. Spacing ranged from 12.7 to 98.2 μm. PDMS with a 10:1 ratio of base to curing agent was thoroughly mixed and degassed, then poured into the microwell array and placed in a 65 °C oven for 4 h (i.e., a soft lithography process). The PDMS was gently peeled from the microwell array to yield the micropillar structures.

### 2.3. Fabrication of Micropillar Array via CNC

In this study, for micropillars with dimensions greater than a hundred microns (i.e, with diameter and depth larger than 300 and 100 μm, respectively), a polymethylmethacrylae (PMMA) plate was milled by a computer numerical control (CNC) machine (Roland EGX-400, Twinsoft Co., New Taipei City, Taiwan) to yield microwells. These molds were subsequently used to obtain micropillar structures via PDMS soft lithography.

### 2.4. Wet Chemical Etching of PDMS Micropillars

A wet chemical etching solution of PDMS was prepared by mixing a 75 wt% aqueous solution of TBAF and 1-Methyl-2-pyrrolidone (NMP) or acetone with a 1:6 volume ratio (about 0.34 M, a relatively high etching rate [31] being intended in this study). Total volume was 42 mL. The etching solution was used immediately after preparation. TBAF was the PDMS etchant, supplying fluoride ions to etch PDMS to form a complex [31]. NMP or acetone served as the organic solvent. The wet chemical etching was performed at room temperature without or with stirring at a rate of 150 rpm. After a certain period of etching time, the etched PDMS micropillar array was cleaned by immersing in DI water. Finally, the sample was dried in a 65 °C oven for 30 min and preserved in a Petri dish. To prevent any drastic change in etching rate, the etchant was replaced with fresh solution after either etching two samples or being placed in an ambient condition for 15 min.

### 2.5. Characterization and Contact Angle Measurements

The PDMS sample was observed using an inverted microscope (Eclipse TE2000-S, NIKON, Tokyo, Japan) and analyzed with a NIKON NIS element. The contact angle and sliding angle of the PDMS surface with micropillars were both measured using a contact angle goniometer (VCA OPTIMA, AST PRODUCTS, Billerica, MA, USA). The PDMS surface with micropillars was placed on the stage. A droplet with designated volume was formed at the syringe tip, and the stage was moved upward till the droplet was in contact with the top of the micropillars. The stage was then moved downward so that the droplet detached from the tip and stayed on top of the micropillars where the contact angle was measured. In cases when the droplet did not detach from the tip, the syringe was slightly tapped to allow the droplet to separate from the tip. To determine the sliding angle, the stage was tilted till the droplet rolled off and the angle was recorded. Three replicates for each measurement were obtained for statistical analysis. A simple peeling test was also conducted; this involved applying a tape to the top of the micropillars which was then peeled off to assess its durability.

## 3. Results and Discussion

### 3.1. Characterization of Etching Micropillar Array

**Figure 1:**
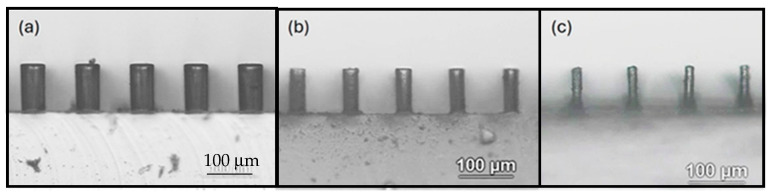
Side-view images of the PDMS cylindrical micropillar array with initial D = 50 μm, H = 100 μm, and W = 50 μm under etching for (**a**) 0 min, (**b**) 2.5 min, (**c**) 5 min. Solvent: NMP. Condition: Stirring.

**Figure 2:**
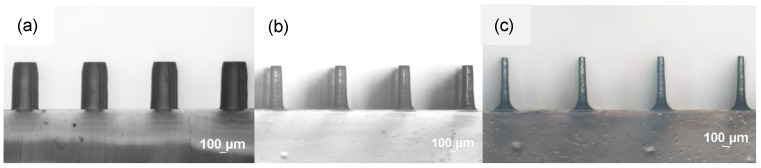
Side-view images of the PDMS cylindrical micropillar array with initial D = 400 μm, H = 700 μm, and W = 600 μm under etching for (**a**) 0 min, (**b**) 17.5 min, (**c**) 32.5 min. Solvent: NMP. Condition: Stirring.

**Figure 3:**
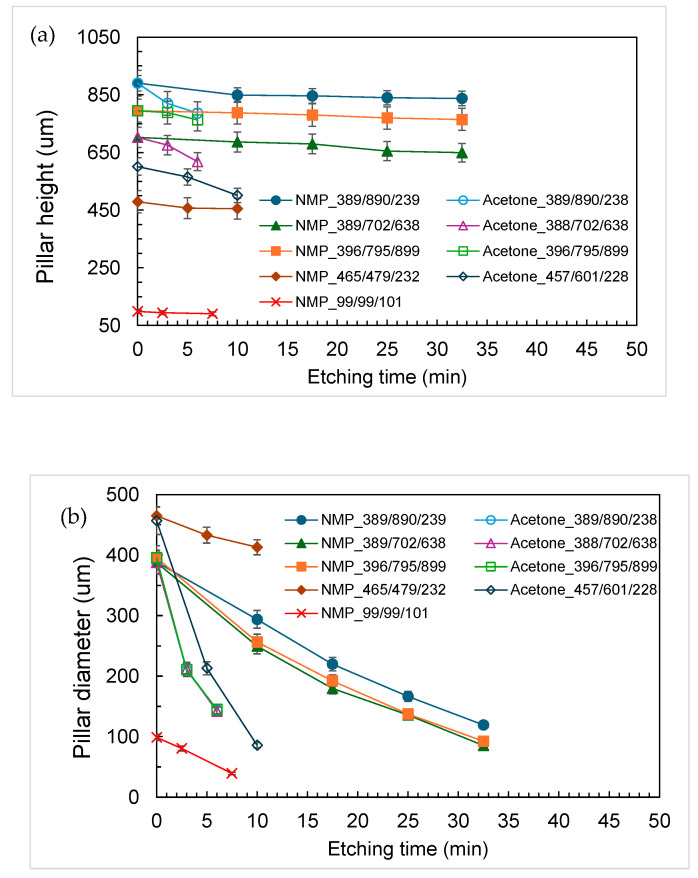
Decreases in (**a**) height and (**b**) diameter in the PDMS cylindrical micropillar array at different etching times. Condition: Stirring.

Figure 1 and Figure 2 show typical time-lapsed images of etching a PDMS micropillar array under stirring. It can be seen that, for micropillars with both smaller (less than 100 μm) and larger (hundreds of microns) dimensions, decreases in heights were minimal. However, substantial decreases in the diameters of micropillars did occur during the etching process. Moreover, the micropillars were isotropically etched in a radial direction so that the cylindrical shape was preserved and micropillars with a very high aspect ratio (up to 8) were constructed. Similar results were obtained when acetone was used as the solvent, except that the etching rate was much higher. Figure 3 shows a comparison of decreases in height and diameter when using NMP and acetone as solvents. The label NMP_389/890/239 refers to “micropillars initially with 389 μm in diameter (D), 890 μm in height (H) and 239 μm in spacing (W) were etched using NMP as solvent”. It can be seen that the etching rates for NMP and acetone in the vertical direction were approximately 1.5 and 12 μm/min, respectively, and approximately 7 and 40 μm/min, respectively, in the radial direction. Why the etching rate is higher when using acetone as solvent, compared when using NMP as solvent, can be reasoned as follows: The etching rate is influenced by the concentration of TBAF and by the type of solvent used, the latter controlling fluoride reactivity, polymer accessibility, and removal/dissolution of disassembled polymer chains [31]. Because the concentration of TBAF is the same in the two systems, it is the type of solvent which plays the major role. Acetone is an aprotic solvent; hence, its use results in higher reactivity of fluoride ions, compared to use of the protic solvent NMP, which suppresses the reactivity of fluoride ions due to H-bonding. Furthermore, acetone has a higher swelling index, which allows the fluoride ions to have better polymer accessibility for etching.

**Figure 4:**
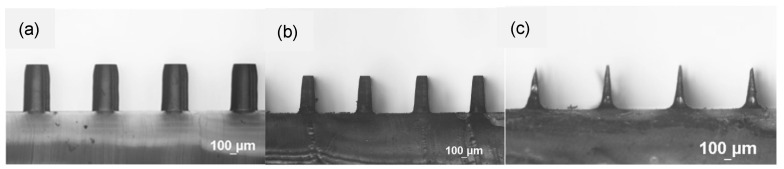
Side-view images of the PDMS cylindrical micropillar array with initial D = 400 μm, H = 700 μm, and W = 650 μm under etching for (**a**) 0 min, (**b**) 17.5 min, (**c**) 40 min. Solvent: NMP. No stirring.

**Figure 5:**
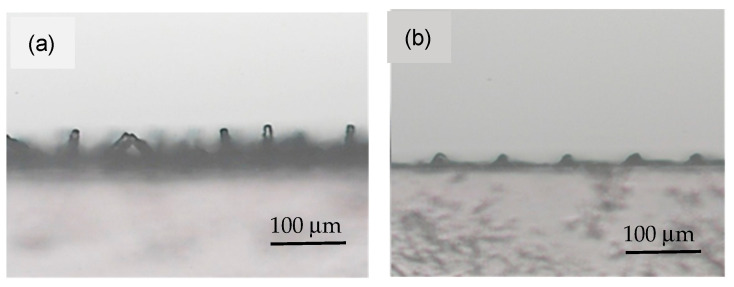
Side-view images of the PDMS cylindrical micropillar array with initial D = 50 μm, H = 100 μm, and (**a**) W = 50 μm, (**b**) W = 30 μm. Solvent: NMP. No stirring. Etching time: 5 min.

**Figure 6:**
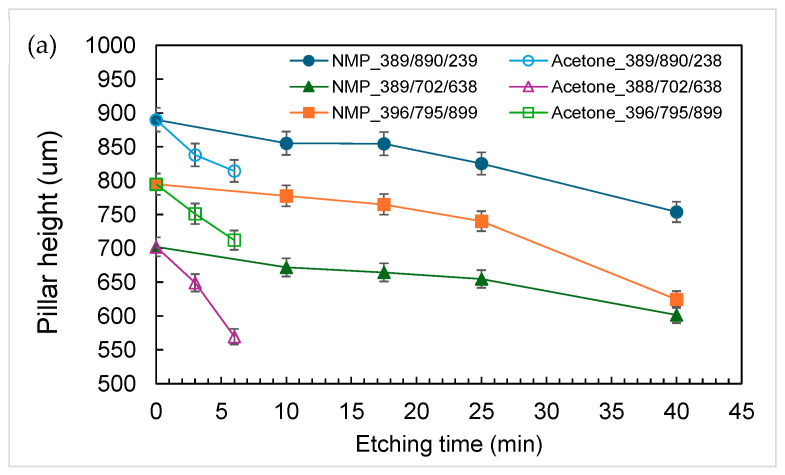

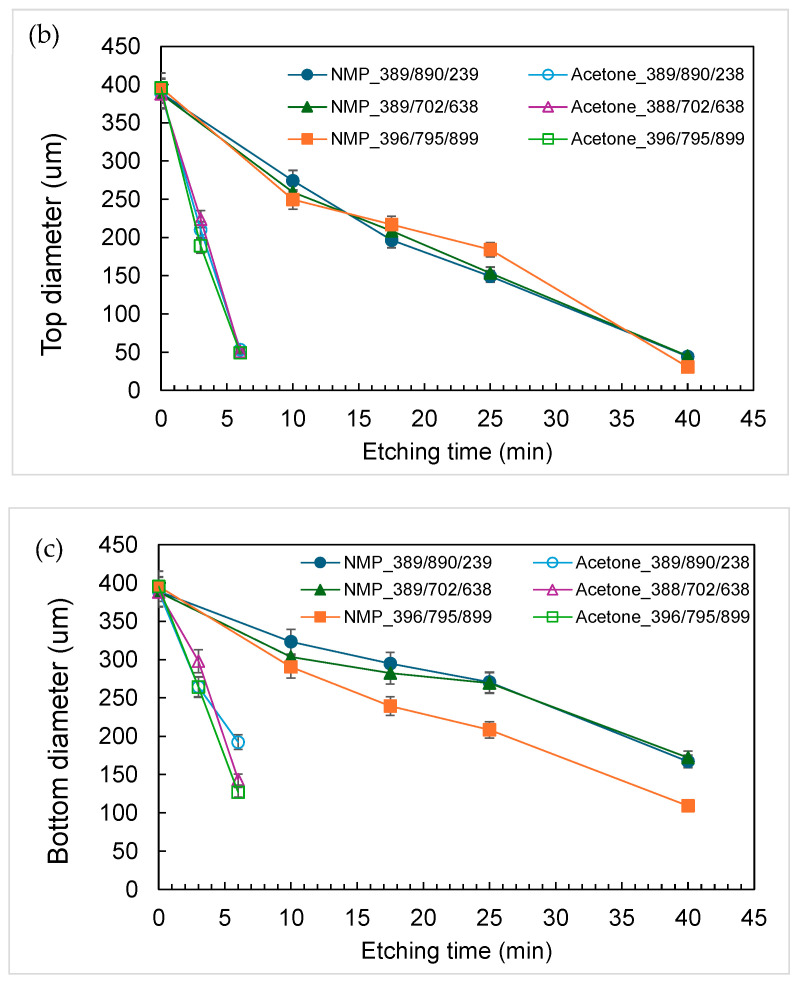
Decreases in (**a**) height, (**b**) top diameter, and (**c**) bottom diameter in the PDMS cylindrical micropillar array at different etching times. Condition: Non-stirring.

When etching was performed without stirring, the micropillars with larger dimensions and spacing (>100 μm) became microneedles, while those with smaller dimensions and spacing (<100 μm) either collapsed or were etched away, as shown in Figure 4 and Figure 5, respectively. The formation of microneedles can be attributed to different radial etching rates near the top and bottom of the micropillars, arising from different etching conditions. That is, the higher etching rate was observed owing to more effective replenishment of etchant and removal of byproduct in contrast to the limited transport occurring near the bottom of the micropillars. Figure 6 shows etching rates in vertical and radial directions. It can be seen that the etching rates for NMP and acetone in the vertical direction are approximately 3.3 and 16 μm/min, respectively, similar to rates under the stirring condition. In the radial direction, the etching rates near the top and bottom of the micropillars for NMP are approximately 8.4 and 5.7 μm/min, respectively, while for acetone they are 57 and 40 μm/min, respectively. Note that the etching rate in the radial direction in both cases is similar to findings in the literature [31], while that in the vertical direction is much lower. The micropillars would not bend after repeated droplet contact and simple testing with tape.

### 3.2. Analysis of Contact Angles, Sliding Angles, and States of Droplets

**Figure 7:**
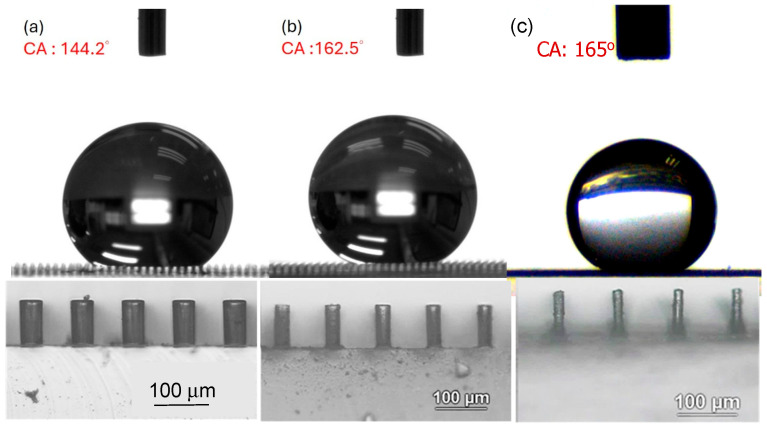
Contact angles of the PDMS cylindrical pillar array with initial D = 50 μm, H = 100 μm, and W = 50 μm for etching times of (**a**) 0 min, (**b**) 2.5 min, (**c**) 5 min.

**Figure 8:**
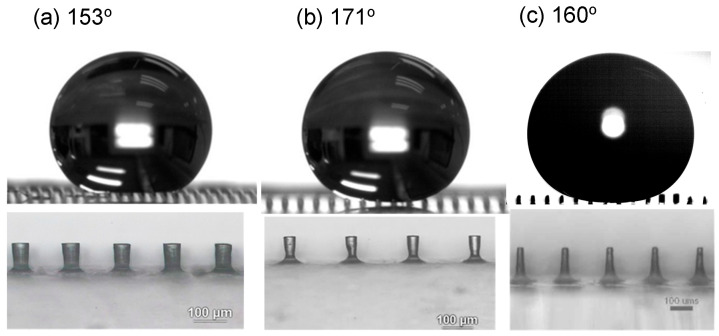
Contact angles of the etched PDMS micropillar array with (D, H, W) values of (**a**) 50, 90, 100 μm; (**b**) 40, 90, 160 μm; (**c**) 30, 150, 170 μm.

**Figure 9:**
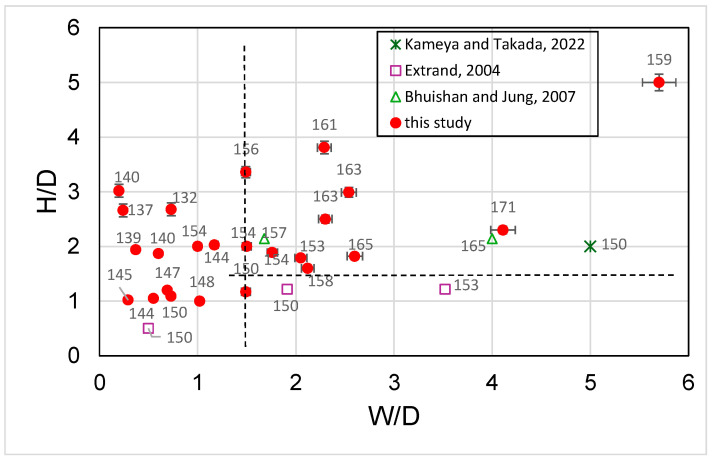
Contact angles at the surface for PDMS micropillars with different W/D and H/D values (droplet volume: 5 μL). (Kameya and Takeda, 2022; Extrand, 2004; Bhuishan and Jung, 2007).

**Figure 10:**
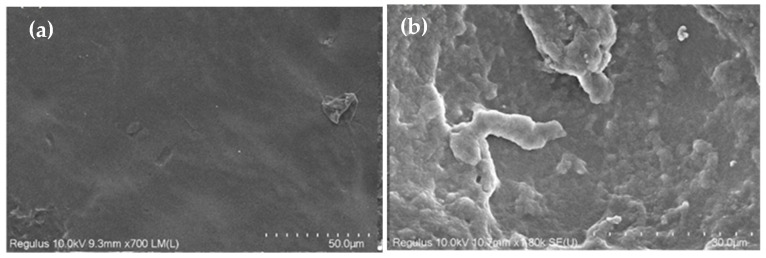

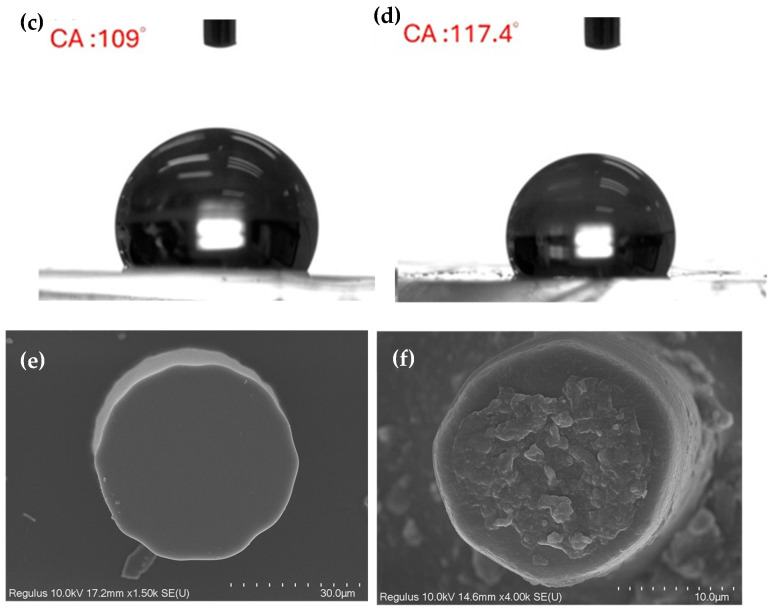
SEM pictures of PDMS surface (**a**) before etching, and (**b**) after 15 min etching. A droplet on (**c**) a pristine PDMS surface, and (**d**) a PDMS surface after 15 min etching. Top surfaces of micropillars (**e**) before etching, and (**f**) after 15 min etching.

**Figure 11:**
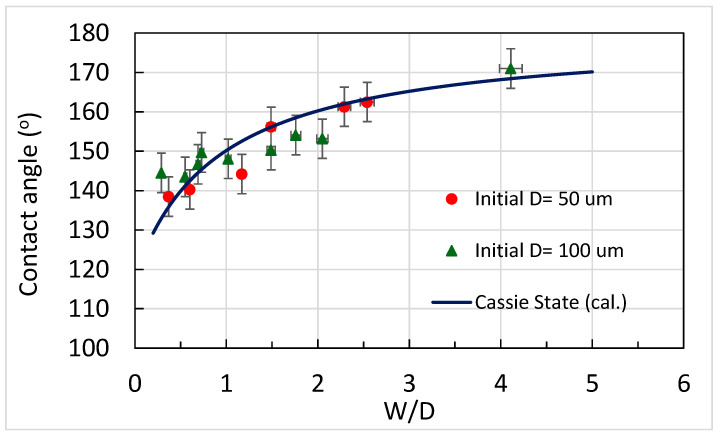
Contact angles on surfaces having micropillars with different W/D values obtained through etching (droplet volume: 5 μL).

**Figure 12:**
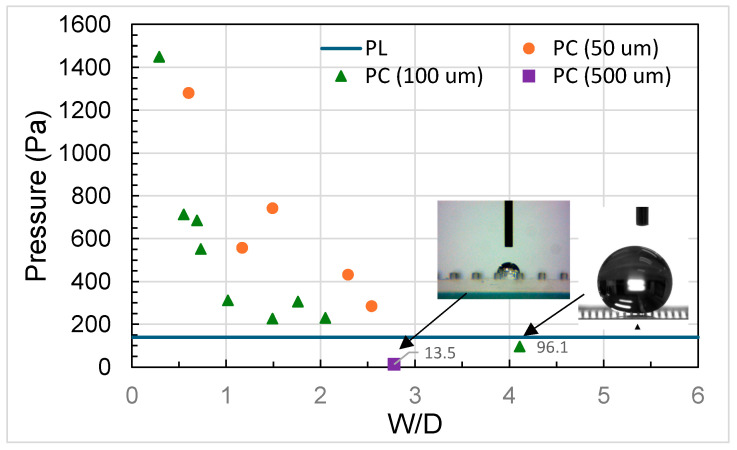
Comparison between Laplace pressure (from 5 μL droplet) and capillary pressure (from micropillars).

**Figure 13:**
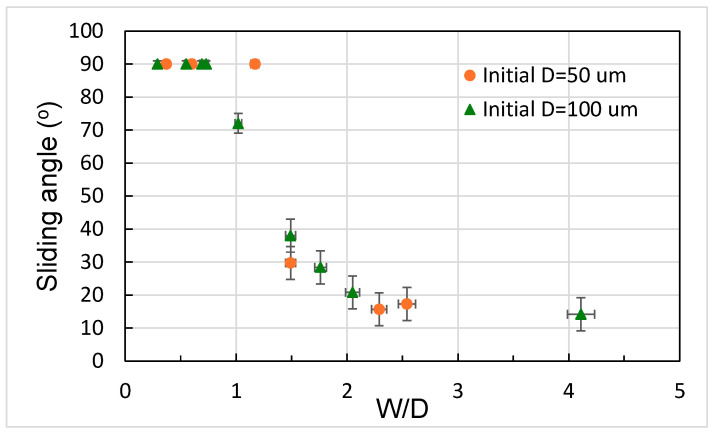
Sliding angles at different W/D values obtained by etching micropillars with different initial diameters. Initial micropillar height: 100 μm.

Figure 7 shows typical measurements of contact angle on each surface obtained at different etching times. It can be seen that the contact angle increased as the etching time increased. This is attributable to a reduction in micropillar diameter (or an increase in spacing between the micropillars) as shown, i.e., a reduction in the solid portion of the composite surface. Figure 8 shows some examples of superhydrophobic surfaces (contact angle greater than 150°) obtained from etching PDMS micropillars initially having smaller or larger dimensions. In Figure 9, H/D is plotted against W/D. H/D is the aspect ratio of the micropillars; this is related to preventing the droplet from reaching the bottom of the micropillars through capillary force [16] and maintaining the droplet in a Cassie or Cassie–Wenzel state. W/D is related to the air portion of the composite surface. According to the following equation [15],

R<R∗~l2h

where *R* is the radius of the droplet, $R^{*}$ is the threshold radius of the droplet that cannot be held by the micropillars, *l* is the spacing between the micropillars, and *h* is the height of the micropillars, it was estimated that $R^{*}$ was between approximately 30 and 800 μm in this study, smaller than the radius of the 5 μL droplet (~1000 μm). That is, the 5 μL droplet was able to be held by the micropillars and thus remain in a Cassie or Cassie–Wenzel state. Moreover, was is found that the surface with PDMS micropillars exhibited a contact angle greater than 150° for a 5 μL droplet when both H/D and W/D were higher than 1.5, a result which can also be found in the literature. Note that the micropillars were made of pristine PDMS without any further surface modification being applied. Nevertheless, it is worthy of mention that, after etching, the PDMS surface morphology changed, as shown in Figure 10a,b. In the figure, it can be seen that the surface was relatively smooth before etching but became a rough, flake-like structure with pores after 15 min of etching. This might be attributed to a slight increase in contact angle (approximately 8°), as shown in Figure 10c,d. The top surface of the micropillars also showed a similar surface morphology after etching (Figure 10e,f). Hence, a larger contact angle for a PDMS surface with micropillars may, to a certain extent, “benefit” from this flak-like structure. On the other hand, the PDMS surface may not bear fluorine atoms to increase hydrophobicity after etching by TBAF. This is because, during PDMS etching, the fluoride ions attack the Si-O-Si bonds in PDMS, yielding fluoride intermediates or terminal silyl fluoride (Si–F) species [31]. Here, two scenarios are possible. One is that fluoride is released back into solution after bond scission and continues to participate in further etching reactions; thus, no permanent Si–F remains at the surface. The other is that fluoride forms silyl fluorides which are prone to hydrolysis [32,33] (if a trace amount of water is present in the etchant, e.g., water content in 75% TBAF solution, minute amounts of water in NMP, or water from rinsing after etching), where silanols and HF are formed, with HF being washed away. Therefore, there should be no persistent chemical modification of the PDMS surface after etching and rinsing. Figure 11 shows changes in contact angle as W/D increased as a result of etching PDMS micropillars. It can be seen that the increase in the contact angle follows the curve calculated using the Cassie–Baxter equation, indicating that increased hydrophobicity can be achieved through etching PDMS micropillars. Further verification of the Cassie–Baxter state of the 5 μL droplet on the surface was carried out using the pressure balance model, in which the transition between the Cassie and Wenzel states is determined by the relative magnitudes of capillary pressure (*P_C_*) and Laplace pressure (*P_L_*) [16]. The equations for capillary and Laplace pressure are as follows:

$$P_{c}=\left[\frac{-4cos\theta_{0}}{{\left(1+\frac{W}{D}\right)}^{2}-1}\right]\frac{\gamma_{LV}}{D}$$

and

$$P_{L}=\frac{2\gamma_{LV}}{R}$$

where $\theta_{0}$ is the contact angle on a smooth surface, $\gamma_{LV}$ is the air–water surface energy, and R is the radius of the droplet. If capillary pressure is greater than Laplace pressure, the droplet remains in a Cassie or Cassie-like state and the bottom surface will not be wetted. The calculated values of *P_C_* and *P_L_* were plotted for comparison, as shown in Figure 12. It can be seen that the *P_L_* value for the 5 μL droplet is approximately 138.7 Pa. As W/D increased, *P_C_* decreased, primarily due to the reduced number of pillars in contact with the droplet. In this study, most of the surfaces with the etched micropillars possessed *P_C_* values higher than *P_L_* values, and the droplets remained in a Cassie or Cassie-like state, in agreement with previous argument. Note that the *P_C_* value for the surface having micropillars with W/D of around 4 was calculated to be around 96, relatively close to the *P_L_* value, and the droplet also remained in a Cassie or Cassie-like state. However, we deliberately fabricated micropillars with W around 1500 μm and D around 500 μm whose *P_C_* value was then calculated to be around 13, much lower than the P_L_ value. Therefore, the Wenzel state of the droplet was observed and the bottom surface was wetted. Figure 13 shows the sliding angles of 5 μL water droplets on the etched micropillar arrays. When W/D was less than 1, the measured angles were 90° and the droplets adhered to the surface. As the W/D ratio reached 1.5, the droplets rolled off the surface and a substantial decrease in the sliding angle to a value around 30° was observed, corresponding to the onset of the larger contact angle, as shown previously. A further increase in W/D led to a decrease in the siding angle, which leveled off at around 10° for a W/D value of around 4.

## 4. Conclusions

In this study, a new method to fabricate a superhydrophobic surface was proposed and demonstrated by etching PDMS micropillars using a process in which different aspect ratios and spacing were achieved. When using acetone as etchant, the etching rate was approximately 7–8 times faster, compared to when NMP was used. With stirring, the micropillars retained a cylindrical shape as etching progressed; under a non-stirring condition, they became needle-like. As the 5 μL droplet was dispensed on the surface, the surface with the etched PDMS micropillars exhibited superhydrophobicity, as both the W/D and H/D values for the micropillars exceeded 1.5. The state of the droplet, i.e., the Cassie or Cassie–Wenzel state, was verified through the threshold radius of the droplet and the pressure balance model. Because different degrees of surface hydrophobicity or superhydrophobicity can be realized through use of a micropillar array, the primary advantage of the proposed method is that these micropillar arrays can be obtained by simply using CNC machining to fabricate microwells, followed by soft lithography in conjunction with PDMS etching. Because the absolute dimensions of the micropillars need not be less than 100 μm, exploitation of CNC micromachining instead of photolithography results in a relatively simple and cost-effective method with fast turnaround times. Moreover, the proposed method provides for structural evolution of micropillars through PDMS etching, enabling various contact angles which correspond to different degrees of hydrophobicity.

## Data Availability

The data presented in this study are available on request from the corresponding author.
